# Prospective neuroimaging and neuropsychological evaluation in adults with newly diagnosed focal epilepsy

**DOI:** 10.1111/epi.18410

**Published:** 2025-05-08

**Authors:** Christophe E. de Bezenac, Nicola Leek, Guleed Adan, Ahmad M. S. Ali, Rajiv Mohanraj, Shubhabrata Biswas, Ronan N. Mcginty, Kieran Murphy, Helen Malone, Gus Baker, Perry Moore, Anthony G. Marson, Simon S. Keller

**Affiliations:** ^1^ Department of Pharmacology and Therapeutics Institute of Systems, Molecular and Integrative Biology, University of Liverpool Liverpool UK; ^2^ The Walton Centre NHS Foundation Trust Liverpool UK; ^3^ Department of Neurology Manchester Centre for Clinical Neurosciences, Salford Royal NHS Foundation Trust Salford UK; ^4^ Liverpool Magnetic Resonance Imaging Centre University of Liverpool Liverpool UK

**Keywords:** neuroimaging, neuropsychology, newly diagnosed focal epilepsy

## Abstract

**Objective:**

Few prospective studies exist on newly diagnosed focal epilepsy (NDFE), a critical period for understanding epilepsy's biology and identifying biomarkers and potential interventions. We report a prospective cohort study in patients with NDFE and age‐, sex‐, and education‐matched healthy controls.

**Methods:**

We recruited 104 patients with NDFE and 45 controls for research‐grade 3 Tesla multi‐modal magnetic resonance imaging (MRI), electroencephalography (EEG), comprehensive neuropsychological testing, and blood biomarker investigations. Baseline clinical, neuroradiological, MRI morphometric, and neuropsychological findings are reported in this article.

**Results:**

Following neuroradiological reporting, MRI was unremarkable in 38% of patients, showed lesions associated with epilepsy in 12%, abnormalities of unknown significance in 49%, and incidental findings in 23%. For controls, these figures were 56%, 7%, 33%, and 16%, respectively. Patients had more white matter hyperintensities, classified as abnormalities of unknown significance, than controls. Reduced bihemispheric frontal lobe cortical thickness and thalamic volumes with moderate effect sizes were observed in patients. Compared to controls, patients scored lower on executive function, processing speed, and visual, delayed, and immediate memory tasks, and higher on depression and anxiety assessments. Cluster analysis identified four distinct patient cognitive profiles, two of which were associated with high levels of anxiety and depression and lower executive function and memory scores.

**Significance:**

Adults with focal NDFE have more MRI‐positive findings than previously reported. Subtle white matter lesions may have clinical significance and a pathophysiological basis in focal epilepsy. Morphometric and neuropsychological changes at epilepsy diagnosis suggest that brain and cognitive alterations are not solely due to chronic epilepsy.

**Key points**

- Investigates neuroimaging and neuropsychological characteristics in newly diagnosed focal epilepsy (NDFE) patients and healthy controls.
- One hundred four NDFE patients and 45 controls underwent 3 Tesla magnetic resonance imaging (MRI), electroencephalography (EEG), neuropsychological tests, and blood biomarker assessments.
- NDFE patients showed more MRI abnormalities, reduced frontal lobe cortical thickness, lower cognitive scores, and higher depression and anxiety levels compared to controls.

## 1 INTRODUCTION

To develop interventions and strategies that will enable us to alter the natural history of epilepsy and improve outcomes for millions of people, it is vital that we understand the biological processes at play in the early stages of the disease; yet the underlying biology of human epilepsy has been studied only rarely from the point of diagnosis. The translation of what we understand in longstanding chronic epilepsy to people with a new diagnosis of epilepsy is confounded by several factors, including uncertainty as to whether the same processes are at play early as well as late in the disease, as well as the chronic effects of seizures and antiseizure medication.^1^ At present we have very limited ability to stratify newly diagnosed patients for important outcomes such as seizure control and cognitive dysfunction, which diminishes our ability to individualize patient management and counseling.^2^, ^3^ A detailed prospective study using novel imaging techniques in newly diagnosed focal epilepsy (NDFE) is required to identify important biomarkers associated with biological processes underpinning the disease, seizure outcome, and comorbidities. However, patients have been prospectively studied only rarely from the time of diagnosis, particularly using combined research grade neuroimaging and cognitive investigations, despite this being a key point in time to understand the underlying biology of epilepsy and to identify potential interventions and biomarkers for seizure and cognitive outcomes.^1^, ^4^

Many people with epilepsy are cognitively impaired at the time of diagnosis. Drug‐naïve patients with NDFE show significant impairments in memory, sustained attention, executive functioning, mental flexibility, and psychomotor speed relative to healthy volunteers.^5^, ^6^, ^7^, ^8^, ^9^, ^10^ One 12‐month follow‐up study revealed that performance on some of these cognitive domains further deteriorate.^11^ Cognitive deficits—which along with spontaneous seizures contribute to impaired quality of life in epilepsy^12^—are therefore not necessarily a result of the chronicity of the disorder, despite that cognitive impairment is likely to be exacerbated by ongoing seizures and the burden of epilepsy.^13^ However, there are currently no insights from existing neuroimaging investigations as to the underlying mechanisms of cognitive dysfunction at the point of epilepsy diagnosis. Given that impairments in brain structural and functional networks—facets of brain organization only amenable to investigation using research grade neuroimaging methods—are becoming increasingly recognized as important markers of seizure phenotypes, treatment outcomes, and cognitive impairment in refractory epilepsy,^14^, ^15^, ^16^ it is crucial that brain networks and other aspects of brain architecture and function not amenable to investigation using standard clinical magnetic resonance imaging (MRI) methods be studied prospectively from the earliest point in human epilepsy using advanced imaging methods.

In order to address this research gap, we have designed a research program that recruited patients with focal NDFE for advanced MRI, resting‐state electroencephalography (EEG), detailed neuropsychological evaluation, and analysis of blood samples with 6 monthly follow‐up to determine patterns of seizure relapse and remission.^17^ The primary objectives of this program of research is to use the detailed and high fidelity neuroimaging, neurophysiological, neuropsychological, and biological data to understand the mechanisms of focal epilepsy and stratify patients according to cognitive phenotypes and antiseizure medication treatment outcomes. The goal of this article is to describe the cohort and present baseline data. We sought to classify neuroradiological findings based on and extending previously published criteria in NDFE,^18^ present basic quantitative MRI findings, and summarize neuropsychological results, exploring interactions between the clinical, diagnostic, imaging, and cognitive data.

## 2 METHODS

This study was approved by the Northwest Liverpool East Research Ethics.

Committee (19/NW/0384) through the Integrated Research Application System (Project ID 260623). The research is sponsored by the University of Liverpool (UoL001449) and UK Health Research Authority approval was provided on 22 August 2019. Recruitment of the first participant was 24 August 2019.

### 2.1 Participants

Patients with focal NDFE were recruited from the Walton Centre National Health Service (NHS) Foundation Trust and Salford Royal (now Northern Care Alliance) NHS Foundation Trust in the UK. Standardized EEG investigations were performed at the respective NHS Trusts. All patients received MRI, neuropsychological evaluation, and blood extraction and storage at the University of Liverpool. The inclusion criteria for patients were diagnosis of focal onset epilepsy by an experienced neurologist based on seizure semiology at one of the two NHS Trusts, between and including the ages 16 and 70 years, and all investigations to be performed within 3 months of diagnosis. We chose not to recruit drug‐naïve patients as this would have substantially limited the recruitment to the study as patients are typically started on antiseizure medication, based on a clinical diagnosis, before investigations (MRI and EEG) are undertaken. Patient exclusion criteria included non‐epileptic seizures, primary generalized seizures, single seizures, provoked seizures only (e.g., alcohol), known inflammatory neurological condition (specifically multiple sclerosis or sarcoidosis), acute symptomatic seizures (e.g., acute brain hemorrhage or brain injury), progressive neurological disease (e.g., known brain tumor), previous neurosurgery, concomitant infection, and any other significant comorbidity (at physicians' discretion). As done previously,^18^ seizures were classified as focal without impairment of awareness (including seizures with observable motor or autonomic phenomena and seizures with subjective sensory and psychic phenomena), focal with impairment of awareness, focal with evolution to bilateral tonic–clonic, or unclassifiable.

We additionally recruited healthy controls matched for age, sex, and education with no history of neurological or psychiatric illness or disease. Control recruitment was staggered prospectively so that each participant had educational attainment similar to that of patients, which we considered to be a significant confounding factor for neuropsychological evaluation. In addition, we monitored age and sex throughout recruitment, making sure that controls were as well matched to patients as possible. All controls were recruited from responses to advertisements at the University of Liverpool and the Walton Centre NHS Foundation Trust and underwent the same investigations as patients. Based on power calculations we intended to recruit 107 patients and 48 controls for MRI, EEG, neuropsychological assessment, and blood sampling.^17^ The calculation was based on the number of patients expected to be seizure‐free and seizure‐active 2 years after recruitment, the number of MRI‐negative/lesional cases, and 10% attrition to follow‐up. All patients underwent follow‐up after neuroimaging and neuropsychological assessment at 6‐month intervals for 2 years to capture the number of seizures experienced in the preceding 6 months and current medications and dosage. Blood was collected for analysis for all participants in lithium‐heparin bottles or serum separator tubes (maximum of 72 mL of blood, 3 × 9 mL vials) by a health care professional trained in phlebotomy. Blood samples have been stored in the University of Liverpool Biobank and will be subject to investigation of peripherally circulating cytokines and markers of cell damage in due course.

### 2.2 Neuroimaging

All participants underwent MRI scanning at the Liverpool Magnetic Resonance Imaging Centre (LiMRIC) at the University of Liverpool using a 3 T Siemens Magnetom Prisma scanner using a 32‐channel head coil. The MRI protocol consisted of structural sequences for screening of incidental findings and identification of lesions, a three‐dimensional (3D) T1‐weighted scan, diffusional kurtosis imaging, resting‐state functional MRI, and a verbal memory functional MRI paradigm adapted from a previous study.^19^ Further information on each MRI sequence is provided in Table S1.

A consultant neuroradiologist (S.B.) with expertise in epilepsy neuroimaging reviewed all patient and healthy control scans for lesions and incidental findings. Only participant age was provided. Neuroradiological features were classified as normal, incidental, or abnormal. Abnormal features were further divided into likely focal epilepsy‐related or unknown relationship to focal epilepsy.^18^ Epilepsy‐related lesions included abnormalities with known potential for epileptogenicity, such as malformations of cortical development, hippocampal sclerosis, foreign tissue lesions, and focal encephalomalacia or gliosis. Abnormalities of unknown relationship to epilepsy included focal and diffuse white matter lesions, diffuse brain atrophy, asymmetric hippocampi, and amygdalae without clear unilateral sclerosis, and historical cerebellar infarcts. For this category, although the brain appears abnormal relative to healthy controls of a similar age, the relationship between the specific abnormality and the underlying epilepsy has not been definitively established.^18^ Unlike previous work,^18^ we classified supratentorial arachnoid cysts as unknown relationship to epilepsy unless ruled out by EEG and seizures localized elsewhere. Possible causal relationships between epilepsy and arachnoid cysts have been suggested.^20^, ^21^, ^22^ All other cysts were classified as incidental. Incidental findings are lesions known to be reported in asymptomatic healthy controls with no previous link to seizures.

For this investigation, we performed volumetry of subcortical, cerebral lobar, and cerebellar regions to determine if patients showed gross brain atrophy compared to controls and whether volumetric alterations were associated with neuropsychological changes. We used FreeSurfer (version 7.1.1) to extract cortical thickness and subcortical volume for brain regions. FreeSurfer is an open‐source neuroimage analysis suite available at https://surfer.nmr.mgh.harvard.edu/. The recon‐all pipeline with default settings was used, including motion correction, averaging of multiple T1 images, removal of non‐brain tissue using a watershed/surface deformation procedure, and segmentation of white matter and subcortical gray matter in Talairach space. FreeSurfer segmentation labels were derived from probabilistic information estimated from expert segmentations of 40 adult brain images. Further technical details of the FreeSurfer process can be found in previous publications.^23^, ^24^ A trained technician visually examined all processed images. No participants were excluded due to artifacts, movement errors, or structural anomalies. Measurements included mean cortical thickness, cortical volume parcellated into 34 bilateral regions of interest (ROIs) as per Desikan et al.,^23^ and total brain volume.

### 2.3 EEG

All patients and controls underwent conventional diagnostic EEG recordings using the international 10–20 system. Each EEG acquisition was evaluated by an expert neurophysiologist for evidence of inter‐ictal epileptiform activity. Each EEG acquisition included resting‐state data, which was identified by a trained clinical EEG technician. These data will be subject to physiological network analysis in due course.^17^

### 2.4 Neuropsychology

All participants underwent computerized neuropsychological assessment using a tailored battery that targeted cognitive domains shown previously to be impacted in patients with NDFE.^5^, ^11^ The battery included components from the Wechsler Memory Scale Fourth Edition (WMS‐IV),^25^ Wechsler Adult Intelligence Scale Fourth Edition (WAIS‐IV),^11^, ^25^ Delis‐Kaplan Executive Function System (D‐KEFS),^26^, ^27^ Patient Health Questionnaire 9 (PHQ‐9),^28^ Generalized Anxiety Disorder 7 (GAD‐7),^29^ Perceived Deficits Questionnaire (PDQ),^30^ and Quality Of Life In Epilepsy (QOLIE) scale.^31^ These assessment tools were used to evaluate auditory memory through story recall and recall of verbal pairs (WMS‐IV), visual memory through the visual reproduction of drawings and the recall of content and spatial features (WMS‐IV), auditory working memory (WAIS‐IV), attention span and executive control (WAIS‐IV), digit span and arithmetic (WAIS‐IV), processing and psychomotor speed (WAIS‐IV), coding and symbol search (WAIS‐IV), finger tapping and visual reaction time (WAIS‐IV), verbal fluency and executive functioning (the Stroop task) (D‐KEFS), mood including depression (PHQ‐9) and anxiety (GAD‐7), perceived cognitive impairment (PDQ), and quality of life (QOLIE). Summary indices were computed for memory (Auditory. Memory, Delayed. Memory, Immediate. Memory, Visual. Memory) and cognition (Working. Memory, Processing Speed, Executive Function). Other represented domains included mood (Depression PHQ9, Anxiety GAD7) and motor function (Finger Tapping RH (right hand), Finger Tapping LH (left hand), Visual RT M (reaction time, mean), Visual.RT.SD (reaction time, standard deviation)).

### 2.5 Statistical analysis

Data analysis was performed using R (version 4.2.0, https://www.R‐project.org/). Tables with associated summary statistics were generated with the R package arsenal (version 3.6.3). Analysis of variance test (ANOVA) was used for continuous variables, chi‐square goodness of fit test was used for categorical or factor variables, and trend test was used for equal distribution of ordered factor variables.

For each neuropsychological feature, we employed a participant‐specific z‐score computation through a resampling approach. This method involved determining the mean and standard deviation (SD) of feature scores for 100 random samples of 10 healthy control participants (ensuring that participants for whom the *z*‐score was being computed was excluded from these samples in the case of controls). The difference between a participant's feature score and the mean of the 100 computed mean values was divided by the mean of the 100 SD values [*z* = (score − mean(100 × mean(scores for 10 controls)))/mean(100 × SD(scores for 10 controls))]. This process resulted in a *z*‐score per feature for both patients and controls.

A *z*‐score threshold greater than 2 (*z*‐score > 2) was applied for features where we anticipated patients to exhibit higher scores (Depression PHQ9, Anxiety GAD7, Visual RT M, Visual.RT.SD). Conversely, a z‐score threshold smaller than 2 (*z*‐score < 2) was used for features where we expected patients to perform worse based on previous work (Finger Tapping RH, Finger Tapping LH, Auditory Memory, Delayed Memory, Immediate Memory, Visual Memory, Working Memory, Processing Speed, Executive Function).^5^, ^6^, ^7^, ^8^, ^9^, ^10^, ^11^ A difference of 2 SD is used frequently to identify abnormal patient scores in clinical practice and research, and has been used to identify abnormal neuropsychological scores in previous studies of NDFE.^5^, ^7^, ^9^ Although fewer studies have used a more liberal threshold of 1 SD,^6^ we explored differences at this level in ancillary analyses.

We performed a cluster analysis of patient neuropsychological *z*‐scores (computed in comparison to the control group) to explore patient profiles. After first scaling the data, we determined the optimal number of clusters using the Elbow method and within‐cluster sum of squares (WCSS), combined with visual inspection of cluster overlap using principal components analysis (Figure 3B). K‐means clustering was used before examining the characteristics of each cluster (neuropsychological patient profile) by computing the means of the patient neuropsychological *z*‐scores within each cluster.

We assessed structural brain difference between patients with NDFE and healthy control subjects using multiple linear regressions (lm function in R). Brain volume was used for subcortical structures and mean thickness for cortical regions. A binary diagnosis indicator (0 = healthy control, 1 = person with focal epilepsy) served as the predictor of interest, and the volume or thickness of a designated brain region constituted the outcome measure. Effect size estimates across all brain regions were computed as Cohen's *d*, adjusting for age, sex, and intracranial volume (ICV) (to control for the association between volume with head size). To address multiple comparisons, we applied a false discovery rate (FDR) correction at *q* = .05.

## 3 RESULTS

### 3.1 Cohort

The coronavirus disease (COVID) pandemic had a significant impact on recruitment. Given the closure of imaging facilities, prioritization of COVID‐related health care, and repurposing of clinical staff, recruitment was delayed by 16 months (Figure S1). We recruited 104 patients with focal NDFE and 45 healthy controls, 3 patients and 3 controls short of our target. There were no significant differences between patients and controls with respect to age, sex, or education, although there was a trend for controls to be older than patients (Table 1). Most patients did not have a familial history of epilepsy, had fewer than 10 seizures at the time of diagnosis, did not have nocturnal seizures, experienced convulsive seizures with loss of awareness, had seizures preceded by an aura, and had no history of febrile seizures, brain infection, head injury, or birth complications (Table 1). Twenty six (25%) patients reported to have experienced 10 or more seizures prior to diagnosis, and three (2.9%) reported more than 100 seizure events. Antiseizure medication information for each patient is provided in Table S2.

**TABLE 1:** Summary of demographic and clinical data.

	Control (*N* = 45)	Patient (*N* = 104)	Total (*N* = 149)	*p* Value
Age				
Mean (SD)	40.752 (15.745)	35.777 (13.526)	37.280 (14.362)	.052
Range	17.205–68.371	16.657–69.228	16.657–69.228	
Sex, *n* (%)				
Male	24 (53.3%)	57 (54.8%)	81 (54.4%)	.868
Female	21 (46.7%)	47 (45.2%)	68 (45.6%)	
Completed high school				
Yes	28 (62.2%)	62 (59.6%)	90 (60.4%)	.765
No	17 (37.8%)	42 (40.4%)	59 (39.6%)	
Ethnicity				
White British	40 (88.9%)	94 (90.4%)	134 (89.9%)	.697
White other	1 (2.2%)	4 (3.8%)	5 (3.4%)	
Multiple	1 (2.2%)	3 (2.9%)	4 (2.7%)	
African	1 (2.2%)	2 (1.9%)	3 (2.0%)	
Asian	2 (4.4%)	1 (1.0%)	3 (2.0%)	
Family history of epilepsy				
No	45 (100.0%)	80 (76.9%)	125 (83.9%)	<.001
Yes	0 (.0%)	24 (23.1%)	24 (16.1%)	
Months since first seizure				
Min		0		
Median		7		
Mean		30.65		
Max		350		
Number of seizures				
N‐Miss		9		
Five or less		61 (64.2%)		
More than five		34 (35.8%)		
Nocturnal seizures				
N‐Miss		0		
Yes		80 (76.9%)		
No		19 (18.3%)		
Sometimes		5 (4.8%)		
Convulsive seizures				
N‐Miss		0		
Yes		73 (70.2%)		
No		24 (23.1%)		
Unsure		7 (6.7%)		
Loss of awareness				
N‐Miss		0		
Yes		61 (58.7%)		
No		19 (18.3%)		
Sometimes		5 (4.8%)		
Unsure		19 (18.3%)		
Seizures with aura				
N‐Miss		0		
Yes		56 (53.8%)		
No		28 (26.9%)		
Sometimes		1 (1.0%)		
Unsure		19 (18.3%)		
Febrile seizures				
N‐Miss		0		
Yes		11 (10.6%)		
No		88 (84.6%)		
Unsure		5 (4.8%)		
History of brain infection				
N‐Miss		0		
Yes		1 (1.0%)		
No		100 (96.2%)		
Unsure		3 (2.9%)		
History of head injury				
N‐Miss		0		
Yes		19 (18.3%)		
No		84 (80.8%)		
Unsure		1 (1.0%)		
Birth complications				
N‐Miss		0		
Yes		21 (20.2%)		
No		79 (76.0%)		
Unsure		4 (3.8%)		
Outcome at 6 months				
N‐Miss		10		
PS		41 (43.6%)		
SF		53 (56.4%)		
Outcome at 12 months				
N‐Miss		27		
PS		32 (41.6%)		
SF		45 (58.4%)		
Outcome at 18 months				
N‐Miss		36		
PS		22 (32.4%)		
SF		46 (67.6%)		
Outcome at 24 months				
N‐Miss		52		
PS		22 (42.3%)		
SF		30 (57.7%)		

Abbreviations: n, number; N‐Miss, number of cases missing; PS, persistent seizures; SD, standard deviation; SF, seizure free.

**FIGURE 1:**
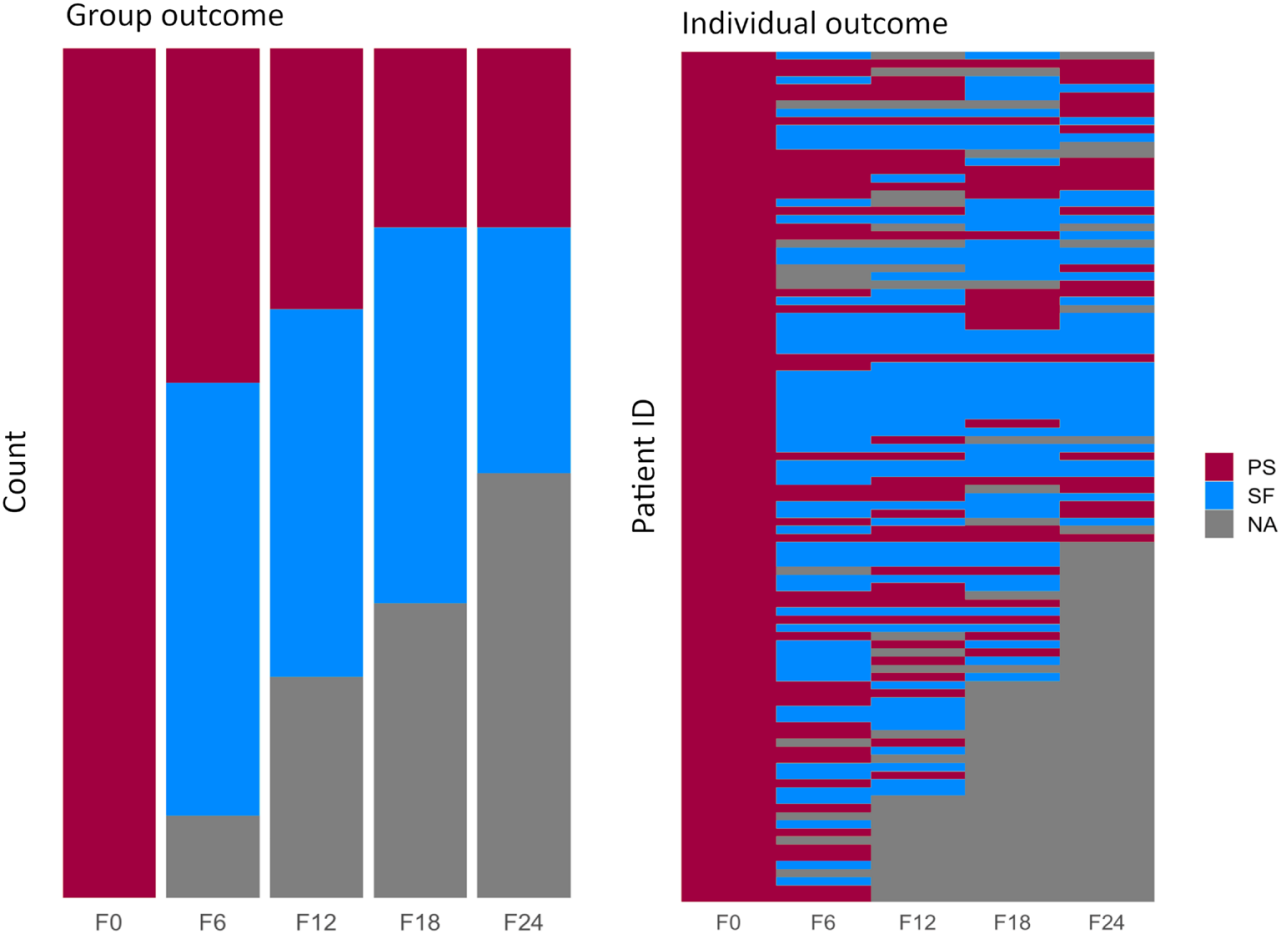
Patient outcome over time. The proportion of patients with NDFE that have persistent seizures (PS) and those that are seizure‐free (SF) for 0 (time of testing), 6‐, 12‐, 18‐, and 24‐month follow‐ups (left). Individual‐level outcomes over time (right). Gray indicates data missing or not yet acquired. NA, data not yet available.

Figure 1 and Table 1 show the recorded post‐treatment outcome for patients in whom these data were available at the time of writing. There were 56.7% of patients (at 6 months), 57.5% (12 months), 66.7% (18 months), and 61.7% (24 months) of patients who did not experience a seizure within the preceding 6 months. Many patients did not have consistent outcomes across each the 6‐month period (Figure 1). Of the 83 patients with 24 months outcome to date (and ignoring missing data), only 37% and 18% were consistently seizure‐free or experienced seizures across every 6‐month period, respectively.

### 3.2 Neuroradiological findings

**FIGURE 2:**
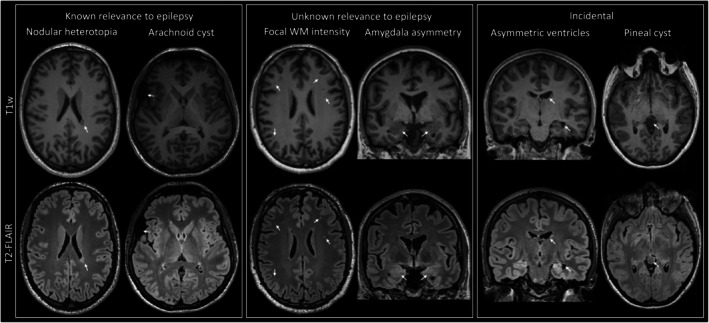
Exemplar MRI‐positive cases across the three lesional categories. Known relevance to epilepsy: Nodular heterotopia of the left supra‐trigonal periventricular region (left) and arachnoid cyst overlying anterior aspect of the right sylvian fissure, abutting the inferior frontal gyrus superiorly and the temporal pole inferiorly (right). Unknown relevance to epilepsy: Multiple focal white matter hypointensities (T1w) and hyperintensities (T2‐FLAIR) (left) and amygdala asymmetry (right; note additional ventricular asymmetry). Incidental: Asymmetric lateral ventricles, including frontal and temporal horns (left) and pineal cyst (right).

Table 2 presents a summary of the neuroradiological findings. Sixty‐one percent of patients had MRI reports classified as abnormal, compared to 44% of controls (*p* = .053). There were 38% of patients with unremarkable MRI features, 12% with lesions of known significance in epilepsy, 49% with abnormalities of unknown significance in epilepsy, and 23% with incidental findings. In comparison, 56% of controls had unremarkable MRI features, 7% had lesions of known significance in epilepsy, 33% had abnormalities of unknown significance in epilepsy, and 16% had incidental findings. Figure 2 provides examples of MRI‐detected lesions classified as epilepsy related, unknown relevance to epilepsy, and incidental.

**TABLE 2:** Neuroradiological and neurophysiological findings.

	Control (*N* = 45)	Patient (*N* = 104)	Total (*N* = 149)	*p* Value
MRI Report Conclusion				
Abnormal	20 (44.4%)	64 (61.5%)	84 (56.4%)	.053
Normal	25 (55.6%)	40 (38.5%)	65 (43.6%)	
EEG Conclusion				
N‐Miss	4	8	12	.638
Abnormal	8 (19.5%)	13 (13.5%)	21 (15.3%)	
Unknown	3 (7.3%)	6 (6.2%)	9 (6.6%)	
Normal	30 (73.2%)	77 (80.2%)	107 (78.1%)	
Epilepsy Related Single Lesion				
Yes	3 (6.7%)	11 (10.6%)	14 (9.4%)	.453
No	42 (93.3%)	93 (89.4%)	135 (90.6%)	
Epilepsy Related Cavernoma				
Yes	1 (2.2%)	0 (.0%)	1 (.7%)	.127
No	44 (97.8%)	104 (100.0%)	148 (99.3%)	
Epilepsy Related Neoplasm				
Yes	0 (.0%)	4 (3.8%)	4 (2.7%)	.182
No	45 (100.0%)	100 (96.2%)	145 (97.3%)	
Epilepsy Related Malformations Of Cortical Development				
Yes	0 (.0%)	3 (2.9%)	3 (2.0%)	.250
No	45 (100.0%)	101 (97.1%)	146 (98.0%)	
Epilepsy Related Heterotopia				
Yes	0 (.0%)	2 (1.9%)	2 (1.3%)	.349
No	45 (100.0%)	102 (98.1%)	147 (98.7%)	
Epilepsy Related Supratentorial Old Stroke				
Yes	0 (.0%)	1 (1.0%)	1 (.7%)	.509
No	45 (100.0%)	103 (99.0%)	148 (99.3%)	
Epilepsy Related Supratentorial Encephalomalacia				
Yes	2 (4.4%)	3 (2.9%)	5 (3.4%)	.627
No	43 (95.6%)	101 (97.1%)	144 (96.6%)	
Unknown Single Lesion				
Yes	15 (33.3%)	51 (49.0%)	66 (44.3%)	.076
No	30 (66.7%)	53 (51.0%)	83 (55.7%)	
Unknown Multiple Lesions				
Yes	1 (2.2%)	15 (14.4%)	16 (10.7%)	.027
No	44 (97.8%)	89 (85.6%)	133 (89.3%)	
Unknown Other				
Yes	0 (.0%)	2 (1.9%)	2 (1.3%)	.349
No	45 (100.0%)	102 (98.1%)	147 (98.7%)	
Unknown Other Supratentorial Hyperintensity				
Yes	1 (2.2%)	3 (2.9%)	4 (2.7%)	.818
No	44 (97.8%)	101 (97.1%)	145 (97.3%)	
Unknown Arachnoid Cyst				
Yes	2 (4.4%)	4 (3.8%)	6 (4.0%)	.865
No	43 (95.6%)	100 (96.2%)	143 (96.0%)	
Unknown Other Supratentorial Neoplasm				
Yes	0 (.0%)	1 (1.0%)	1 (.7%)	.509
No	45 (100.0%)	103 (99.0%)	148 (99.3%)	
Unknown Asymmetric Amygdala				
Yes	0 (.0%)	5 (4.8%)	5 (3.4%)	.135
No	45 (100.0%)	99 (95.2%)	144 (96.6%)	
Unknown Asymmetric Hippocampus				
Yes	0 (.0%)	2 (1.9%)	2 (1.3%)	.349
No	45 (100.0%)	102 (98.1%)	147 (98.7%)	
Unknown Hyperintensity In Amygdala Or Hippocampus				
Yes	0 (.0%)	4 (3.8%)	4 (2.7%)	.182
No	45 (100.0%)	100 (96.2%)	145 (97.3%)	
Unknown Cortical Atrophy				
Yes	2 (4.4%)	4 (3.8%)	6 (4.0%)	.865
No	43 (95.6%)	100 (96.2%)	143 (96.0%)	
Unknown White Matter Hyperintensities				
Yes	11 (24.4%)	41 (39.4%)	52 (34.9%)	.078
No	34 (75.6%)	63 (60.6%)	97 (65.1%)	
Incidental Single Lesion				
Yes	7 (15.6%)	24 (23.1%)	31 (20.8%)	.299
No	38 (84.4%)	80 (76.9%)	118 (79.2%)	
Incidental Multiple Lesions				
Yes	0 (.0%)	5 (4.8%)	5 (3.4%)	.135
No	45 (100.0%)	99 (95.2%)	144 (96.6%)	
Incidental Other				
Yes	4 (8.9%)	4 (3.8%)	8 (5.4%)	.210
No	41 (91.1%)	100 (96.2%)	141 (94.6%)	
Incidental Isolated Developmental Venous Anomaly				
Yes	2 (4.4%)	2 (1.9%)	4 (2.7%)	.382
No	43 (95.6%)	102 (98.1%)	145 (97.3%)	
Incidental Infratentorial Old Stroke				
Yes	0 (.0%)	6 (5.8%)	6 (4.0%)	.100
No	45 (100.0%)	98 (94.2%)	143 (96.0%)	
Incidental Infratentorial Encephalomalacia				
Yes	0 (.0%)	1 (1.0%)	1 (.7%)	.509
No	45 (100.0%)	103 (99.0%)	148 (99.3%)	
Incidental Non Arachnoid Cyst				
Yes	1 (2.2%)	6 (5.8%)	7 (4.7%)	.347
No	44 (97.8%)	98 (94.2%)	142 (95.3%)	
Incidental Enlarged Or Asymmetric Ventricles				
Yes	0 (.0%)	4 (3.8%)	4 (2.7%)	.182
No	45 (100.0%)	100 (96.2%)	145 (97.3%)	
Incidental Cavum Septum Or Vergae				
Yes	0 (.0%)	2 (1.9%)	2 (1.3%)	.349
No	45 (100.0%)	102 (98.1%)	147 (98.7%)	
Incidental Dilated Perivascular Spaces				
Yes	0 (.0%)	3 (2.9%)	3 (2.0%)	.250
No	45 (100.0%)	101 (97.1%)	146 (98.0%)	

Single lesions of significance in epilepsy were found in 11% of patients, but also in 7% of controls. Neoplasms were observed in 4% of patients and no controls (*p* = .18). The next most common epilepsy‐related finding was supratentorial encephalomalacias (3% of patients and 4% of controls) and malformations of cortical development (3% of patients and no controls). White matter hyperintensities of unknown significance in epilepsy were observed in 49% of patients, compared to 24% of controls (*p* = .08). Similarly, single lesions of unknown significance were found in 49% of epilepsy patients and 33% of controls (*p* = .08), whereas 14% of patients and 2% of controls had multiple unknown lesions (*p* = .03). Asymmetry of the amygdala and hippocampus was identified in 4% and 2% of patients, respectively, and in 0% of controls (*p* = .13, *p* = .35). Single lesions not thought to be related to epilepsy (i.e., incidental) were observed in 23% of patients and 16% of controls (*p* = .3), and 5% of patients (no controls) had multiple incidental lesions (*p* = .13). Six percent of patients (no controls) had an infratentorial old stroke, and 4% (no controls) had enlarged or asymmetric ventricles (*p* = .18). The most frequent incidental lesions were non‐arachnoid cysts (patients 5.8%, controls 2.2%, *p* = .35), historical infratentorial infarct (patients 5.8%, controls 0%, *p* = .10), isolated developmental venous anomalies (patients 1.9%, controls 4.4%, *p* = .38), and enlarged or asymmetric lateral ventricles (patients 3.8%, controls 0%, *p* = .18) (Table 2).

### 3.3 Quantitative structural MRI

**FIGURE 3:**
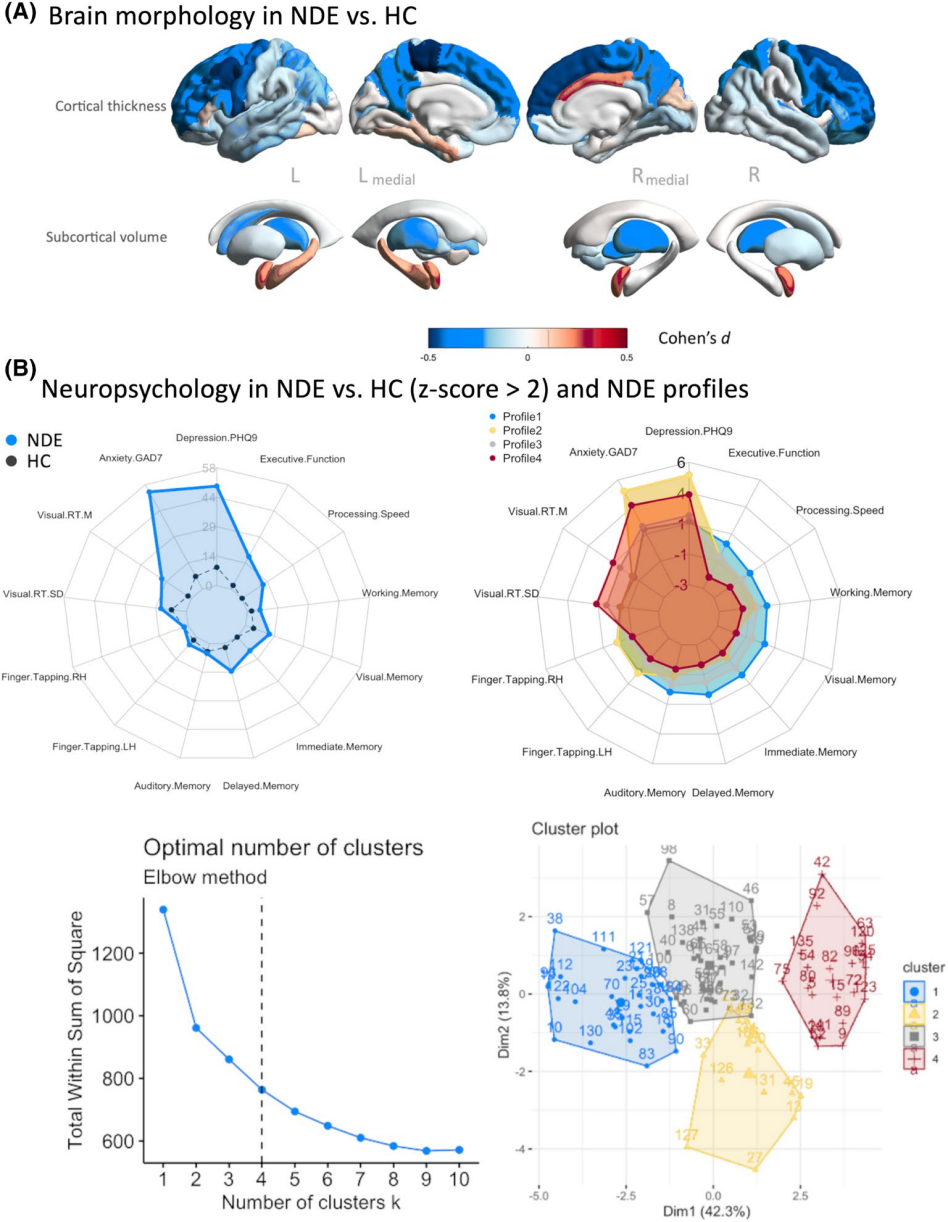
Quantitative MRI and neuropsychological differences between patients and controls. (A) MRI brain morphometrics. Differences displayed as Cohen's *d* in regional cortical thickness (top) and subcortical volumes (bottom) in patients with NDFE compared to healthy controls. Blue indicates larger values in controls, red indicates larger values in patients. (B) Neuropsychology radar plot indicating the percentage of patients with NDFE with *z*‐scores greater than 2 SD for each neuropsychological feature (blue) compared to healthy controls (black) (top left). Optimal number of clusters using the Elbow method and within‐cluster sum of squares (WCSS) and cluster overlap using PCA (bottom left). Patient Profiles 1–4 of neuropsychological *z*‐scores (right).

After correction for multiple comparisons, there were no significant differences in subcortical volumes, regional cortical thickness, or cerebellar volumes between patients and controls (Table S3). Furthermore, there were no significant differences in the same MRI metrics between patients who experienced a first seizure within 12 months of diagnosis (*n* = 67) and patients who had a first seizure beyond 12 months (*n* = 38) (Table S4). Given that we expected morphometric alterations in the early stages of focal epilepsy to be subtle, we also explored differences without FDR correction, as the direction and pattern of findings provide information about overall trends that may become more pronounced with disease progression. Results indicated no differences in subcortical or cerebellar volume but decreased cortical thickness predominantly in the bilateral frontal regions. In the left hemisphere, this included precentral (*t* = 2.429, *p* = .016), rostral middle frontal (*t* = 2.117, *p* = .036), and superior frontal (*t* = 2.00, *p* = .047) cortex. In the right hemisphere, caudal middle frontal (*t* = 2.287, *p* = .024), paracentral (*t* = 2.015, *p* = .046), pars opercularis (*t* = 2.045, *p* = .043), rostral middle frontal (*t* = 2.207, *p* = .029), and superior frontal (*t* = 2.541, *p* = .012) cortical regions were thinner in patients. Figure 3A shows the Cohen's *d* effect sizes of regional volume and cortical thickness differences and illustrates reduced cortical thickness predominantly in hemispheric frontal lobe regions in patients compared to controls.

Given that some brain changes are likely to be related to the currently unknown seizure focus that varies across patients, we also computed the percentage of brain regions with negative *z*‐scores (*Z* < 0) (i.e., volume/thickness reduction compared to controls) across the whole brain (irrespective of where alterations were located). Patients showed an average of 20% of brain regions with reduced volume across the whole brain compared to 16% of regions in controls. We fitted a linear model (estimated using ordinary least squares) to predict percentage of brain regions with negative z‐scores with Group, after controlling for age, sex, and ICV. We found a non‐significant trend, with patients showing increased percentage (beta = 4.35, 95% confidence interval [CI]: −0.77 to 9.48, *t*(147) = 1.68, *p* = .095; standardised beta = 0.30, 95% CI: −0.05 to 0.65). The model explains a statistically not significant and very weak proportion of variance (*R* ^2^ = .02, *F*(1, 147) = 2.82, *p* = .095, adj. *R* ^2^ = .01). The model's intercept, corresponding to Group = Control, is at 16.14 (95% CI: 11.85– 20.42, *t*(147) = 7.45, *p* < .001).

### 3.4 Neuropsychological findings

Table 3 presents the neuropsychological findings in our cohort of patients and controls. Patients had significantly lower scores on tests of executive function, processing speed, visual memory, delayed memory, and immediate memory compared to controls. Patients also had significantly higher scores on measures of depression and anxiety. The discrepancy between patients and controls on tests of depression and anxiety exceeded that of executive function, processing speed and memory. This difference was reflected in the proportion of patients calculated to exceed 2 SD on individual tests, as illustrated in Figure 3B. Forty‐nine percent and 54% of patients had scores on tests of depression and anxiety greater than 2 SD compared to 9% and 7% in controls, respectively. This contrasted to 18%, 12%, 12%, 14%, and 9% of patients and 2%, 0%, 4%, 2%, and 0% of controls in tasks of executive function, processing speed, visual memory, delayed memory, and immediate memory, respectively. Similar to quantitative MRI results, there were no statistically significant differences in neuropsychological variables between patients who experienced a first seizure within 12 months of diagnosis and those who had a first seizure beyond 12 months (Table S5). Using a more liberal threshold of 1 SD, which is occasionally adopted, there were changes to the variables that were significantly different between patients and controls (contrast Table 3 and Table S6; Figure S2). Depression (*p* < .001), anxiety (*p* < .001), and visual reaction time (*p* = .02) remained different, visual memory (*p* = .001) and finger tapping (*p* = .02) became significant, and executive function (*p* = .07), processing speed (*p* = .09), delayed memory (*p* = .07), and immediate memory (*p* = .10) were no longer statistically different. There were no differences between patients within and beyond 12 months since first seizure when a 1 SD threshold was adopted (Table S7).

**TABLE 3:** Neuropsychological characteristics of patients with NDFE and controls: *Z*‐score > 2.

	Control (*N* = 45)	Patient (*N* = 104)	Total (*N* = 149)	*p* Value
Depression PHQ9				
No	41 (91.1%)	53 (51.0%)	94 (63.1%)	<.001
Yes	4 (8.9%)	51 (49.0%)	55 (36.9%)	
Anxiety GAD7				
No	42 (93.3%)	48 (46.2%)	90 (60.4%)	<.001
Yes	3 (6.7%)	56 (53.8%)	59 (39.6%)	
Executive Function				
No	44 (97.8%)	85 (81.7%)	129 (86.6%)	.008
Yes	1 (2.2%)	19 (18.3%)	20 (13.4%)	
Visual RT M				
No	44 (97.8%)	86 (82.7%)	130 (87.2%)	.011
Yes	1 (2.2%)	18 (17.3%)	19 (12.8%)	
Processing Speed				
No	45 (100.0%)	91 (87.5%)	136 (91.3%)	.013
Yes	0 (.0%)	13 (12.5%)	13 (8.7%)	
Delayed Memory				
No	44 (97.8%)	89 (85.6%)	133 (89.3%)	.027
Yes	1 (2.2%)	15 (14.4%)	16 (10.7%)	
Immediate Memory				
No	45 (100.0%)	95 (91.3%)	140 (94.0%)	.042
Yes	0 (.0%)	9 (8.7%)	9 (6.0%)	
Visual Memory				
No	43 (95.6%)	91 (87.5%)	134 (89.9%)	.134
Yes	2 (4.4%)	13 (12.5%)	15 (10.1%)	
Working Memory				
No	44 (97.8%)	98 (94.2%)	142 (95.3%)	.347
Yes	1 (2.2%)	6 (5.8%)	7 (4.7%)	
Visual RT SD				
No	42 (93.3%)	92 (88.5%)	134 (89.9%)	.364
Yes	3 (6.7%)	12 (11.5%)	15 (10.1%)	
Finger Tapping LH				
No	44 (97.8%)	99 (95.2%)	143 (96.0%)	.461
Yes	1 (2.2%)	5 (4.8%)	6 (4.0%)	
Finger Tapping RH				
No	44 (97.8%)	102 (98.1%)	146 (98.0%)	.905
Yes	1 (2.2%)	2 (1.9%)	3 (2.0%)	
Auditory Memory				
No	43 (95.6%)	99 (95.2%)	142 (95.3%)	.923
Yes	2 (4.4%)	5 (4.8%)	7 (4.7%)	

Cluster analysis revealed four patient profiles (Figure 3B): Profiles 1 and 3 show subgroups of patients closest to controls with lower depression and anxiety scores and, particularly in the case of Profile 1, higher executive function, processing speed, and memory (working, visual, immediate, delayed, and auditory). In contrast, Profiles 2 and 4 represented subgroups with higher anxiety and depression (particularly Profile 2) as well as lower executive function, processing speed, and memory. Profile 4 also shows higher reaction time (mean and SD) and lower right‐ and left‐hand finger tapping, possible suggesting a sensorimotor dysfunction in this patient subgroup.

No correlations between brain morphology and neuropsychology features in patients with NDFE were significant after FDR correction for multiple comparisons (q‐value threshold of .05). However, the overall pattern of results including uncorrected significance levels are shown in Figure S3. The direction of the association (red = positive correlation, blue = negative correlation) and strength of the association (size of connections, level of significance) are indicated. Positive relationships can be seen between visual memory and superior frontal and parietal regions, and between working memory and volume of the amygdala and temporal poles. We also found immediate, auditory, and delayed memory to be negatively correlated with the caudate (left/right) and the right pericalcarine area in patients.

## 4 DISCUSSION

Our neuroradiological findings indicate an unexpectedly high number of positive MRI findings in both groups, with a greater proportion of lesions with unknown relevance to epilepsy in patients, particularly focal white matter hyperintensities. Quantitative neuroimaging data suggested reduced cortical thickness in the frontal lobe and thalamus in patients compared to controls. Neuropsychological data indicated significantly greater depression and anxiety scores in patients, with moderate impairments in executive function, processing speed, and memory related to cortical and subcortical morphometric data. Finally, we observed significant changes in seizure outcome status at 6‐month intervals in the patients who had multiple follow‐up data.

### 4.1 Neuroimaging findings

A recent study reported that 59.3% of adolescents and adults with focal NDFE were MRI negative, 18.7% had lesions considered epilepsy related, 17% had abnormalities with unknown relationship to epilepsy, and 5% had incidental findings.^19^ Our findings were 38%, 12%, 49%, and 23%, respectively. The largest difference was a higher proportion of patients with abnormalities with unknown relationship to epilepsy, driven by white matter alterations on T2–fluid‐attenuated inversion recovery (FLAIR) images. A significant strength of our study was the inclusion of healthy controls for comparison. The number of patients with white matter abnormalities was greater than controls, suggesting that these findings could have relevance for adult‐onset epilepsy. White matter hyperintensities represent macrostructural brain damage associated with multiple underlying causes and implications^32^ and could be considered potentially clinically significant. A leading cause of focal white matter lesions is small vessel ischemia, which has been associated with focal‐onset seizures in some studies.^33^ The higher incidence of white matter changes in patients could not be attributed to an aging cohort given that our controls were slightly older than our patients. We are unsure as to the reason for the discrepancy in the reported proportion of patients with white matter abnormalities between our study and the aforementioned previous study,^18^ but this could be due to the different age distribution of cohorts (ours was 17–69 years, previous study was 11–65 years) or methodological factors such as our use of identical MRI sequences, a single high‐end 3 T scanner and a single epilepsy‐experienced neuroradiologist for evaluation all patients and controls. Our report of MRI‐positive findings in our healthy controls (44%) is higher than reported previously,^34^ which is driven by the increased incidence of white matter anomalies (24%). Whether our relatively high pick‐up rate of white matter incidental lesions/lesions of unknown relationship to epilepsy is due to our advanced research‐dedicated MRI protocol requires further investigation.

Our results are consistent with previous reports of a low proportion of adults with NDFE presenting with known epileptogenic lesions, including hippocampal sclerosis and malformations of cortical development.^18^, ^35^ We did not observe a single case with clear hippocampal sclerosis (although hippocampal asymmetry was reported in one patient). Unlike previously,^18^ we classified supratentorial arachnoid cysts as related to epilepsy (unless there was clear evidence to suggest an alternative cause) as these lesions have been associated with epilepsy in previous studies.^20^, ^21^, ^22^ However, the inclusion of these lesions as potentially epileptogenic did not increase the incidence of epilepsy‐related lesions in our cohort compared to the previous study.^18^

After stringent correction for multiple comparisons, we report no statistically significant changes in regional compartmental brain volume in patients with NDFE compared to controls, although moderate effect sizes were observed for bilateral thalamic volume reduction and especially frontal lobe cortical thinning. Few studies employ quantitative MRI approaches in NDFE. We have previously reported thalamic volume reduction in an independent heterogeneous group of patients with non‐lesional focal NDFE using manual MRI measurements,^36^ and thalamic atrophy is a known feature of longstanding focal and generalized epilepsy.^37^ Hippocampal atrophy is not a common finding in NDFE.^38^ There are fewer studies examining cortical alterations in NDFE, although one longitudinal study in patients with established focal epilepsy reported that the most pronounced cortical degeneration was observed in the first 5 years after the onset of seizures.^39^ Despite our cohort's clinical heterogeneity, there is a clear pattern of gray matter changes in the frontal lobe that may indicate subtle atrophy in early focal epilepsy. Gross brain alterations are more subtle in focal NDFE compared to chronic focal epilepsy, which may be due to a combination of heterogeneous patient groups and limited insights into architectural and network abnormalities not visible on routine MRI data. Some brain alterations in epilepsy may be established by the time of the first seizure and could result from genetic factors, developmental processes, occult insult, or other mechanisms of epileptogenesis.

### 4.2 Neuropsychological findings

It is well established that chronic epilepsy is associated with cognitive and psychiatric deficits that are dependent on the semiology, etiology, and phenotype of epilepsy disorder,^40^, ^41^ and that deficits may deteriorate progressively as uncontrolled seizures continue.^41^, ^42^ It is equally well established that cognitive and affective deficits are present in drug‐naïve patients with NDFE, particularly in the domains of memory, sustained attention, executive functioning, mental flexibility and psychomotor speed.^5^, ^6^, ^7^, ^8^, ^9^, ^10^ Our work supports these findings. A previous study reported that impairments in attention and executive functions were seen in 49.4% and memory deficits in 47.8% of patients with drug‐naïve patients with NDFE, and recommended cognitive screening in new‐onset epilepsies,^43^ which is not currently embedded in clinical evaluation pathways. In patients with new‐onset seizures, speed/executive function (35%) and memory (29%) were reported to be the most impacted cognitive domains and that cognitive dysfunction may be a predictor of seizure recurrence.^44^ Other work has reported that administration of anti‐seizure medications has minimal impact on cognitive impairment in NDFE^45^ and it was reported that cognitive impairment exists despite preserved general intelligence in the early stages of epilepsy.^46^ Most patients underreport cognitive problems in the early stages of epilepsy as the new onset of seizures causes the greatest concern, but early neuropsychological monitoring could improve individual medical care throughout a lifetime lived with epilepsy.^43^ Prospective longitudinal studies in people with NDFE and new‐onset epilepsy may help disentangle the relationships between seizures and comorbidities,^47^ which is what we hope our program of research will help achieve.

Cluster analysis results have indicated that there may be distinct neuropsychological profiles in people with NDFE. Neuropsychological taxonomic groupings have been reported in patients with chronic epilepsy. For example, patients with chronic temporal lobe epilepsy have been reported to have three distinct cognitive profiles based on cluster analysis, including minimally impaired (47%); memory impaired (24%); and memory, executive, and speed impaired (29%).^40^, ^48^ Latent profile analysis has revealed distinct cognitive phenotypes in patients with semiologically and etiologically similar refractory temporal lobe epilepsy and that patients with a multi‐dominant cognitive deficit profile have a worse clinical trajectory.^49^ In patients with chronic temporal lobe epilepsy, distinct patterns of white matter structural alterations are associated with different cognitive phenotypes.^50^ We identified two profiles (2 and 4) characterized by greater anxiety and depression and increased impairment on tasks of executive function and memory in patients with NDFE. It will be interesting to determine how these profiles evolve into neuropsychological profiles observed in chronic epilepsy, how newly diagnosed taxonomic groupings relate to pharmacoresistance and clinical trajectories, and whether diverse brain structural and functional abnormalities underlie distinct cognitive/affective phenotypes through the early stages of the disorder.

In children with new‐onset epilepsy, mild diffuse cognitive impairment was not related to MRI morphometric total cerebral or lobar tissue volumes.^51^ This may suggest that the neurobiological substrate of cognitive impairment in NDFE exists beyond what can be inferred from examining gross brain morphology. With respect to neuroradiological findings, it has been previously reported that lesional NDFE is associated with more deficits in attention and executive functions compared to non‐lesional NDFE, and worse memory performance was related to generalized tonic–clonic seizures.^43^ Other work has reported significant changes in delayed visual memory and executive functioning in patients with newly diagnosed temporal lobe epilepsy and no structural lesions.^52^ We report trends between various neuropsychological and mood measures and brain morphometric variables (Figure 3C), which require further investigation, particularly with respect to progressive changes in cognitive phenotypes. Longitudinal imaging and neuropsychological studies will be important in determining whether progressive deterioration in cognition^11^ is related to pathological progressive brain changes through the early stages of epilepsy. Moreover, given that psychiatric problems including depression may antedate the diagnosis of epilepsy in adults,^53^ it will be important to monitor brain‐psychiatric coupling throughout early epilepsy.

### 4.3 Future work

Given that neuroradiological and volumetric MRI data provide limited insights into seizure and cognitive status in NDFE, it is important to phenotype and stratify patients using imaging methods that are sensitive to the brain's microstructural and network architecture. Imaging markers of pharmacoresistance will likely be microstructural, functional, or metabolic. Brain connectivity and network approaches have successfully prognosticated patients with refractory epilepsy,^14^, ^15^, ^16^ and similar approaches should be explored to predict pharmacoresistance and understand cognitive dysfunction mechanisms. We are in a novel position, having acquired imaging data suitable for investigating microstructural brain architecture and structural and functional brain networks in patients with NDFE, and we aim to determine their mechanistic and clinical utility in early epilepsy stages. Furthermore, given the high proportion of patients with white matter hyperintense lesions, future work should quantitatively examine the relationship between these lesions and neuropsychological deficits in our sample, given the previously reported association in patients with different neurological disorders.^32^

## 5 CONCLUSION

The prevalence of MRI‐negative cases in patients with NDFE is lower than previously thought. A significant contributor is the prevalence of white matter hyperintensities, and the relationship between these and focal epilepsy onset requires further investigation. Heterogeneous cohorts of patients with NDFE do not present with strong group‐wise changes in brain morphometry, but moderate alterations are present in the frontal lobe and thalamus. Neuropsychological and mood impairments are present at diagnosis, some related to gross morphometric brain alterations. This suggests that cognitive and psychiatric assessments should be considered in the early stages of epilepsy to support cognitive rehabilitation strategies.

## AUTHOR CONTRIBUTIONS

Cd.B.: acquisition and analysis of data and drafting of the manuscript and figures. N.L.: acquisition and analysis of data. G.A.: acquisition and analysis of data. A.A.: acquisition and analysis of data. R.M.: conception and design of the study and acquisition and analysis of data. S.B.: acquisition and analysis of data. R.M.G.: acquisition and analysis of data. K.M.: acquisition and analysis of data. H.M.: acquisition and analysis of data. G.B.: conception and design of the study. P.M.: conception and design of the study. A.M.: conception and design of the study, acquisition and analysis of data, and drafting of the manuscript. S.K.: conception and design of the study and drafting of the manuscript and figures.

## CONFLICT OF INTEREST STATEMENT

The authors have no conflicts of interest.

## ETHICAL APPROVAL

We confirm that we have read the Journal's position on issues involved in ethical publication and affirm that this report is consistent with those guidelines. This study was approved by the Northwest Liverpool East Research Ethics Committee (19/NW/0384) through the Integrated Research Application System (Project ID 260623). The research is sponsored by the University of Liverpool (UoL001449), and UK Health Research Authority approval was provided on 22 August 2019.

## Supporting information


Figure S1.

Figure S2.

Figure S3.

Table S1.

Table S2.

Table S3.

Table S4.

Table S5.

Table S6.

Table S7.

## Data Availability

The data that support the findings of this study are available via the corresponding author, upon reasonable request.
