# Supplementary figures and images for: Prospective neuroimaging and neuropsychological evaluation in adults with newly diagnosed focal epilepsy
Source: Epilepsia. 2025 May 8;66(8):2864–80. doi: 10.1111/epi.18410 (PMC12371684; doi:10.1111/epi.18410)

## Slide 1
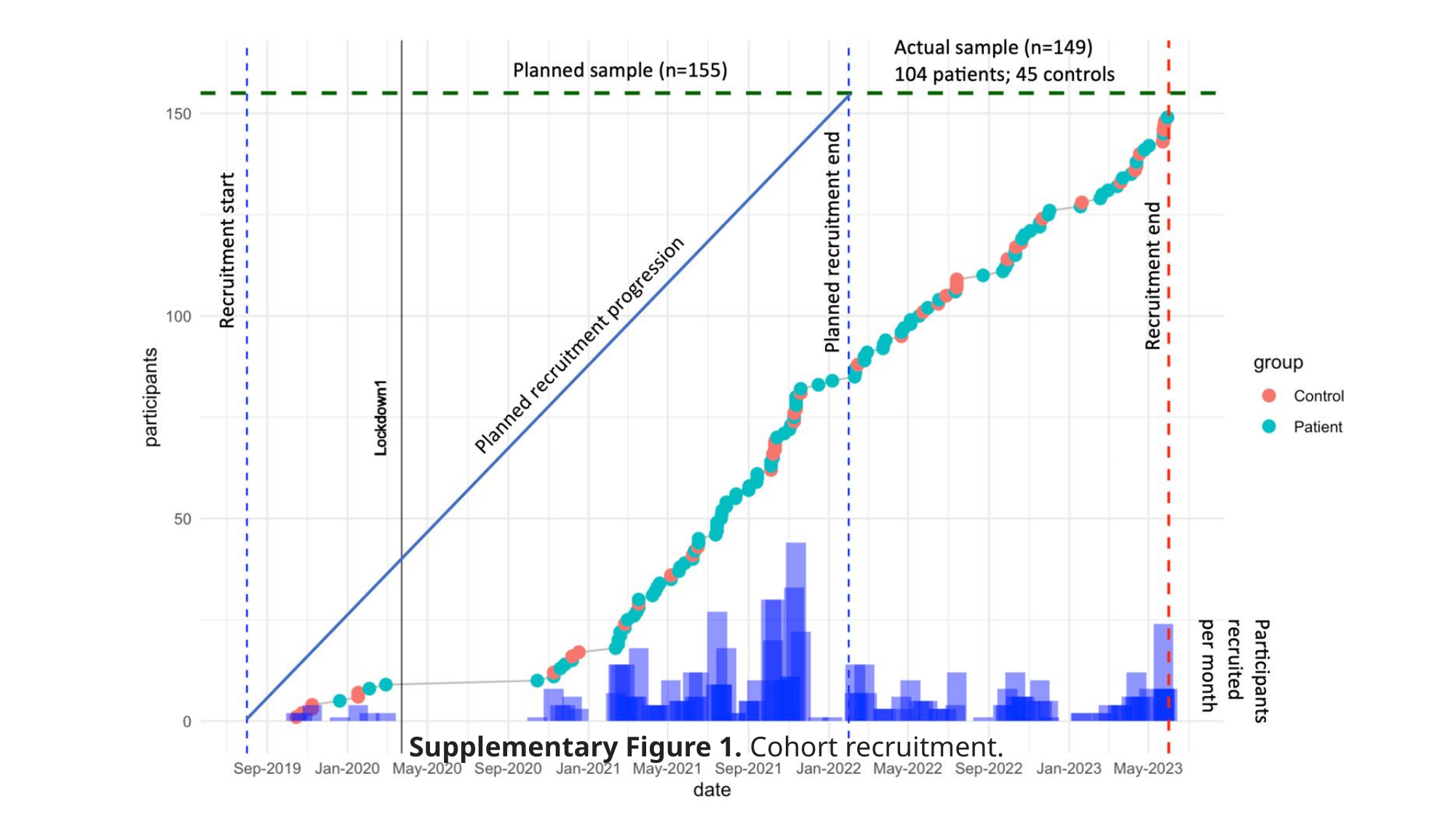

Supplementary Figure 1. Cohort recruitment.

Supplement: Supplementary file 1 — Figure S1. [file EPI-66-2864-s002.pptx]

## Slide 1
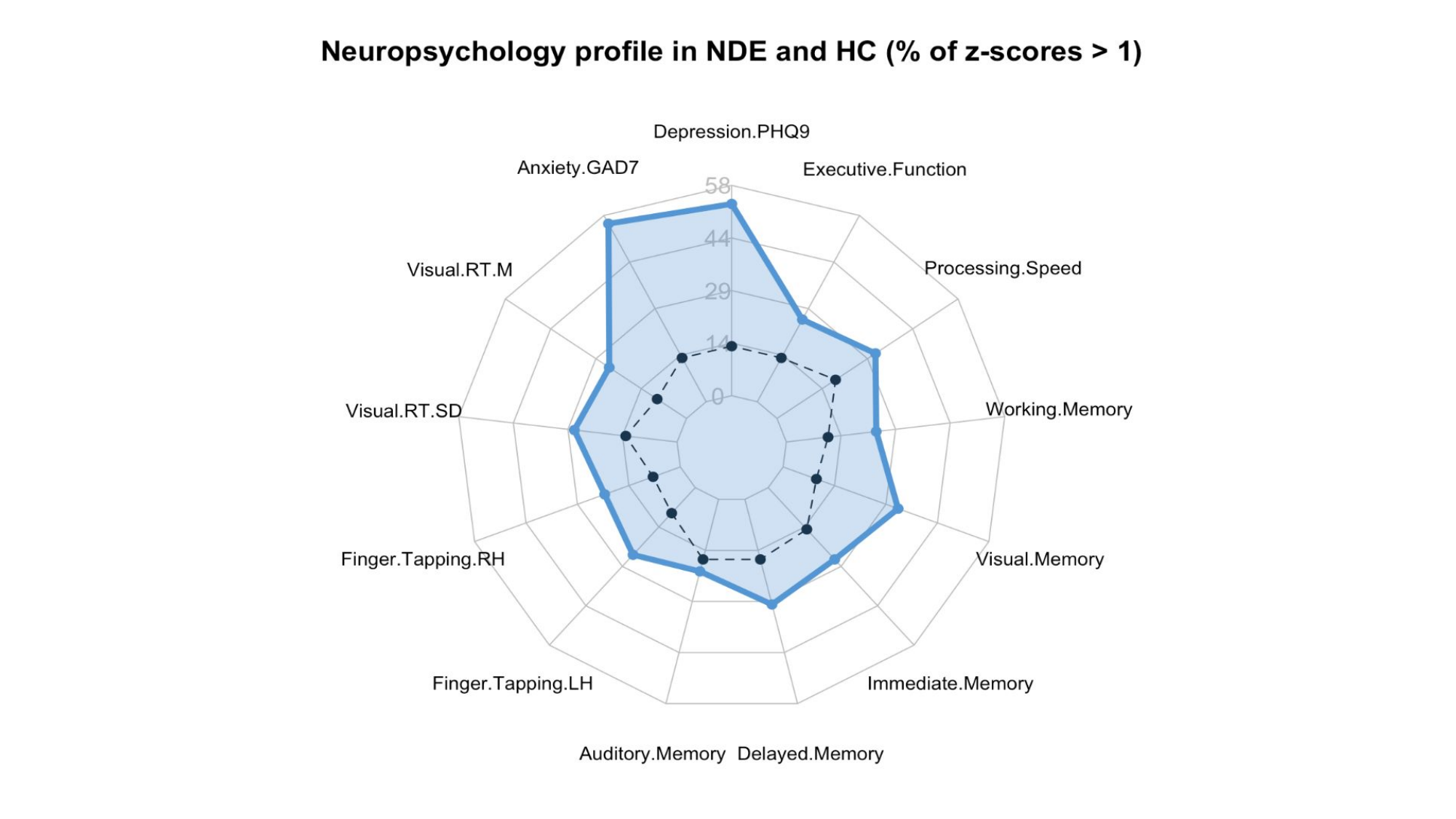

Supplement: Supplementary file 2 — Figure S2. [file EPI-66-2864-s004.pptx]

## Slide 1
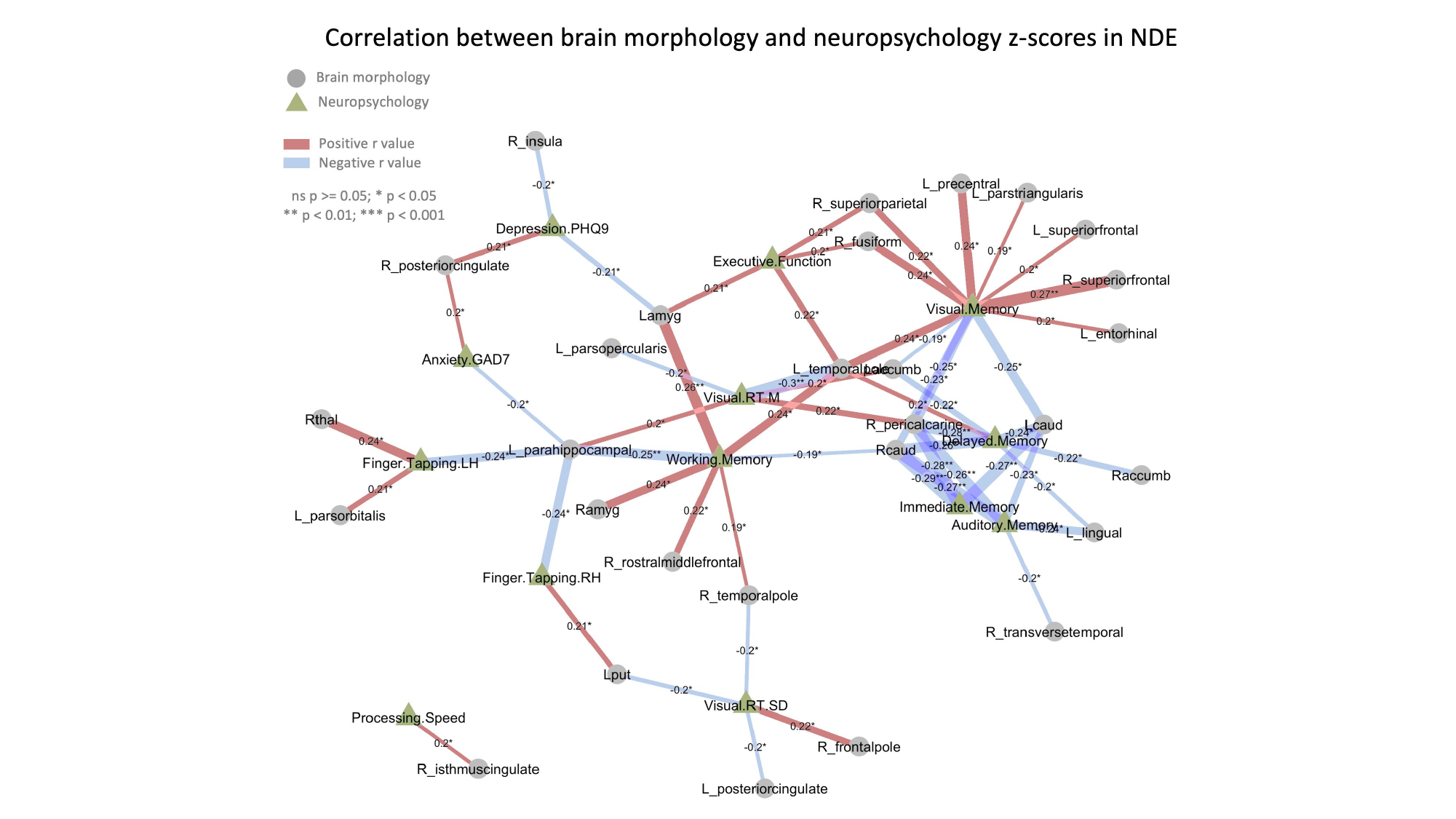

Supplement: Supplementary file 3 — Figure S3. [file EPI-66-2864-s008.pptx]
