# Supplementary material for: Prospective neuroimaging and neuropsychological evaluation in adults with newly diagnosed focal epilepsy
Source: Epilepsia. 2025 May 8;66(8):2864–80. doi: 10.1111/epi.18410 (PMC12371684; doi:10.1111/epi.18410)
Supplement: Supplementary file 8 — Table S5. [file EPI-66-2864-s006.doc]

**Supplementary Table 5.** Neurocognitive characteristics of EPINET patients with less and more than 12 months since first seizure: z-score > 2

|  | <12 (N=65) | >12 (N=39) | Total (N=104) | p value |
| --- | --- | --- | --- | --- |
| **Depression.PHQ9** |  |  |  | 0.960 |
| No | 33 (50.8%) | 20 (51.3%) | 53 (51.0%) |  |
| Yes | 32 (49.2%) | 19 (48.7%) | 51 (49.0%) |  |
| **Anxiety.GAD7** |  |  |  | 0.685 |
| No | 29 (44.6%) | 19 (48.7%) | 48 (46.2%) |  |
| Yes | 36 (55.4%) | 20 (51.3%) | 56 (53.8%) |  |
| **Executive.Function** |  |  |  | 0.326 |
| No | 55 (84.6%) | 30 (76.9%) | 85 (81.7%) |  |
| Yes | 10 (15.4%) | 9 (23.1%) | 19 (18.3%) |  |
| **Visual.RT.M** |  |  |  | 0.894 |
| No | 54 (83.1%) | 32 (82.1%) | 86 (82.7%) |  |
| Yes | 11 (16.9%) | 7 (17.9%) | 18 (17.3%) |  |
| **Processing.Speed** |  |  |  | 0.939 |
| No | 57 (87.7%) | 34 (87.2%) | 91 (87.5%) |  |
| Yes | 8 (12.3%) | 5 (12.8%) | 13 (12.5%) |  |
| **Delayed.Memory** |  |  |  | 0.719 |
| No | 55 (84.6%) | 34 (87.2%) | 89 (85.6%) |  |
| Yes | 10 (15.4%) | 5 (12.8%) | 15 (14.4%) |  |
| **Immediate.Memory** |  |  |  | 0.787 |
| No | 59 (90.8%) | 36 (92.3%) | 95 (91.3%) |  |
| Yes | 6 (9.2%) | 3 (7.7%) | 9 (8.7%) |  |
| **Visual.Memory** |  |  |  | 0.939 |
| No | 57 (87.7%) | 34 (87.2%) | 91 (87.5%) |  |
| Yes | 8 (12.3%) | 5 (12.8%) | 13 (12.5%) |  |
| **Working.Memory** |  |  |  | 0.128 |
| No | 63 (96.9%) | 35 (89.7%) | 98 (94.2%) |  |
| Yes | 2 (3.1%) | 4 (10.3%) | 6 (5.8%) |  |
| **Visual.RT.SD** |  |  |  | 0.751 |
| No | 58 (89.2%) | 34 (87.2%) | 92 (88.5%) |  |
| Yes | 7 (10.8%) | 5 (12.8%) | 12 (11.5%) |  |
| **Finger.Tapping.LH** |  |  |  | 0.287 |
| No | 63 (96.9%) | 36 (92.3%) | 99 (95.2%) |  |
| Yes | 2 (3.1%) | 3 (7.7%) | 5 (4.8%) |  |
| **Auditory.Memory** |  |  |  | 0.407 |
| No | 61 (93.8%) | 38 (97.4%) | 99 (95.2%) |  |
| Yes | 4 (6.2%) | 1 (2.6%) | 5 (4.8%) |  |
