# Supplementary material for: Prospective neuroimaging and neuropsychological evaluation in adults with newly diagnosed focal epilepsy
Source: Epilepsia. 2025 May 8;66(8):2864–80. doi: 10.1111/epi.18410 (PMC12371684; doi:10.1111/epi.18410)
Supplement: Supplementary file 9 — Table S6. [file EPI-66-2864-s007.doc]

**Supplementary Table 6.** Neurocognitive characteristics of patients and controls: z-score > 1.

|  | Control (N=45) | Patient (N=104) | Total (N=149) | p value |
| --- | --- | --- | --- | --- |
| **Depression.PHQ9** |  |  |  | < 0.001 |
| No | 38 (84.4%) | 39 (37.5%) | 77 (51.7%) |  |
| Yes | 7 (15.6%) | 65 (62.5%) | 72 (48.3%) |  |
| **Anxiety.GAD7** |  |  |  | < 0.001 |
| No | 38 (84.4%) | 36 (34.6%) | 74 (49.7%) |  |
| Yes | 7 (15.6%) | 68 (65.4%) | 75 (50.3%) |  |
| **Executive.Function** |  |  |  | 0.067 |
| No | 38 (84.4%) | 73 (70.2%) | 111 (74.5%) |  |
| Yes | 7 (15.6%) | 31 (29.8%) | 38 (25.5%) |  |
| **Visual.RT.M** |  |  |  | 0.019 |
| No | 40 (88.9%) | 74 (71.2%) | 114 (76.5%) |  |
| Yes | 5 (11.1%) | 30 (28.8%) | 35 (23.5%) |  |
| **Processing.Speed** |  |  |  | 0.086 |
| No | 35 (77.8%) | 66 (63.5%) | 101 (67.8%) |  |
| Yes | 10 (22.2%) | 38 (36.5%) | 48 (32.2%) |  |
| **Delayed.Memory** |  |  |  | 0.074 |
| No | 36 (80.0%) | 68 (65.4%) | 104 (69.8%) |  |
| Yes | 9 (20.0%) | 36 (34.6%) | 45 (30.2%) |  |
| **Immediate.Memory** |  |  |  | 0.100 |
| No | 37 (82.2%) | 72 (69.2%) | 109 (73.2%) |  |
| Yes | 8 (17.8%) | 32 (30.8%) | 40 (26.8%) |  |
| **Visual.Memory** |  |  |  | 0.001 |
| No | 40 (88.9%) | 65 (62.5%) | 105 (70.5%) |  |
| Yes | 5 (11.1%) | 39 (37.5%) | 44 (29.5%) |  |
| **Working.Memory** |  |  |  | 0.054 |
| No | 39 (86.7%) | 75 (72.1%) | 114 (76.5%) |  |
| Yes | 6 (13.3%) | 29 (27.9%) | 35 (23.5%) |  |
| **Visual.RT.SD** |  |  |  | 0.041 |
| No | 38 (84.4%) | 71 (68.3%) | 109 (73.2%) |  |
| Yes | 7 (15.6%) | 33 (31.7%) | 40 (26.8%) |  |
| **Finger.Tapping.LH** |  |  |  | 0.019 |
| No | 40 (88.9%) | 74 (71.2%) | 114 (76.5%) |  |
| Yes | 5 (11.1%) | 30 (28.8%) | 35 (23.5%) |  |
| **Finger.Tapping.RH** |  |  |  | 0.024 |
| No | 41 (91.1%) | 78 (75.0%) | 119 (79.9%) |  |
| Yes | 4 (8.9%) | 26 (25.0%) | 30 (20.1%) |  |
| **Auditory.Memory** |  |  |  | 0.590 |
| No | 36 (80.0%) | 79 (76.0%) | 115 (77.2%) |  |
| Yes | 9 (20.0%) | 25 (24.0%) | 34 (22.8%) |  |
