# Supplementary material for: Prospective neuroimaging and neuropsychological evaluation in adults with newly diagnosed focal epilepsy
Source: Epilepsia. 2025 May 8;66(8):2864–80. doi: 10.1111/epi.18410 (PMC12371684; doi:10.1111/epi.18410)
Supplement: Supplementary file 10 — Table S7. [file EPI-66-2864-s010.doc]

**Supplementary Table 7.** Neurocognitive characteristics of patients with less and more than 12 months since first seizure: z-score > 1.

|  | No (N=39) | Yes (N=65) | Total (N=104) | p value |
| --- | --- | --- | --- | --- |
| **Depression.PHQ9** |  |  |  | 0.794 |
| No | 14 (35.9%) | 25 (38.5%) | 39 (37.5%) |  |
| Yes | 25 (64.1%) | 40 (61.5%) | 65 (62.5%) |  |
| **Anxiety.GAD7** |  |  |  | 0.287 |
| No | 16 (41.0%) | 20 (30.8%) | 36 (34.6%) |  |
| Yes | 23 (59.0%) | 45 (69.2%) | 68 (65.4%) |  |
| **Executive.Function** |  |  |  | 0.543 |
| No | 26 (66.7%) | 47 (72.3%) | 73 (70.2%) |  |
| Yes | 13 (33.3%) | 18 (27.7%) | 31 (29.8%) |  |
| **Visual.RT.M** |  |  |  | 0.737 |
| No | 27 (69.2%) | 47 (72.3%) | 74 (71.2%) |  |
| Yes | 12 (30.8%) | 18 (27.7%) | 30 (28.8%) |  |
| **Processing.Speed** |  |  |  | 0.074 |
| No | 29 (74.4%) | 37 (56.9%) | 66 (63.5%) |  |
| Yes | 10 (25.6%) | 28 (43.1%) | 38 (36.5%) |  |
| **Delayed.Memory** |  |  |  | 0.523 |
| No | 27 (69.2%) | 41 (63.1%) | 68 (65.4%) |  |
| Yes | 12 (30.8%) | 24 (36.9%) | 36 (34.6%) |  |
| **Immediate.Memory** |  |  |  | 0.661 |
| No | 28 (71.8%) | 44 (67.7%) | 72 (69.2%) |  |
| Yes | 11 (28.2%) | 21 (32.3%) | 32 (30.8%) |  |
| **Visual.Memory** |  |  |  | 0.272 |
| No | 27 (69.2%) | 38 (58.5%) | 65 (62.5%) |  |
| Yes | 12 (30.8%) | 27 (41.5%) | 39 (37.5%) |  |
| **Working.Memory** |  |  |  | 0.955 |
| No | 28 (71.8%) | 47 (72.3%) | 75 (72.1%) |  |
| Yes | 11 (28.2%) | 18 (27.7%) | 29 (27.9%) |  |
| **Visual.RT.SD** |  |  |  | 0.870 |
| No | 27 (69.2%) | 44 (67.7%) | 71 (68.3%) |  |
| Yes | 12 (30.8%) | 21 (32.3%) | 33 (31.7%) |  |
| **Finger.Tapping.LH** |  |  |  | 0.146 |
| No | 31 (79.5%) | 43 (66.2%) | 74 (71.2%) |  |
| Yes | 8 (20.5%) | 22 (33.8%) | 30 (28.8%) |  |
| **Auditory.Memory** |  |  |  | 0.260 |
| No | 32 (82.1%) | 47 (72.3%) | 79 (76.0%) |  |
| Yes | 7 (17.9%) | 18 (27.7%) | 25 (24.0%) |  |
